# Detection of S-Metolachlor in Surface Water near Cornfields Using pH-Sensitive *Green* Molecularly Imprinted Polymers

**DOI:** 10.3390/molecules31060932

**Published:** 2026-03-11

**Authors:** Dominika Rapacz-Kinas, Katarzyna Smolińska-Kempisty, Agnieszka Urbanowska, Joanna Wolska

**Affiliations:** 1 Department of Process Engineering and Technology of Polymer and Carbon Materials, Wroclaw University of Science and Technology, Wybrzeże Wyspiańskiego 27, 50-370 Wrocław, Poland; 2 Department of Water and Wastewater Management and Waste Technology, Wroclaw University of Science and Technology, Wybrzeże Wyspiańskiego 27, 50-370 Wrocław, Poland

**Keywords:** micropollutants detection, sorption, desorption, surface water, smart polymers

## Abstract

In this study, *core–shell* molecularly imprinted polymers (CS-MIP) were utilized for the detection of the herbicide S-metolachlor in surface water samples, collected from a river and pond that are in the proximity of cornfields. The study revealed that no traces of herbicide were detected in the samples that were analyzed. The collected water samples were treated with membrane filtration—microfiltration and ultrafiltration. The adsorption isotherms were fitted using the Langmuir, Freundlich, Dubinin–Radushkevich, and Scatchard models. This indicated that the Scatchard model is the most appropriate for CS-MIP. The data obtained from the kinetic study were analyzed using the pseudo-first-order and pseudo-second-order models, as well as Fick’s second law. For CS-MIP, the most suitable model was determined to be the particle diffusion model, while for *core–shell* non-imprinted polymers (CS-NIP), the film diffusion model was identified as the limiting step. A method for the desorption of S-metolachlor from the pH-sensitive sorbent bed has been developed, thereby enabling the material to be reused. The optimum eluent from the multicomponent solution was determined to be a 30% aqueous ethanol solution with a pH of approximately 9. This solution effectively removed the majority of contaminants, with the exception of S-metolachlor, which was retained within polymer pores.

## 1. Introduction

Plant protection products perform a crucial function in the protection of crops against diseases, insects, and weeds [1]. Herbicides are of significant importance in present-day agriculture, as they control weeds and ensure the sustainable productivity of crops. Nevertheless, the employment of herbicides has given rise to concerns regarding their contamination, which poses a grave threat to the environment, biodiversity, and food safety [2]. At present, the global consumption of pesticides is estimated to be in excess of 4.10 million tonnes, with herbicides accounting for 47.50% of this total. The utilization of pesticides has undergone a marked increase, with a growth of over 40% observed in the last two decades [2,3,4]. Aquatic ecosystems are defined as complex biotic systems that include a variety of organisms. The impact of herbicides on such ecosystems is twofold: firstly, as a direct result of their physiological effects on organisms, and secondly, through indirect ecological interactions between species [5].

S-metolachlor is a chloroacetamide herbicide that is extensively utilized on a global scale due to its low cost and wide spectrum of activity, particularly in the cultivation of corn crops [6,7]. On an international level, the excessive application of S-metolachlor and metolachlor has resulted in significant environmental contamination issues [7]. S-metolachlor was classified as moderately toxic—Class III [6]. It was found that the majority of S-metolachlor and metolachlor residues are retained within the soil matrix. Owing to their heightened water solubility and reduced adsorption properties, these substances are susceptible to leaching into surface water and groundwater [7]. Following the transfer of S-metolachlor and metolachlor from soil to aquatic systems, there is an observed increase in their half-life, which is prolonged from 15 to 70 days to 97–200 days. This effect is indicative of an increased persistence of the pollutants [8].

Numerous research studies have been carried out across the globe that have focused on the elevated levels of herbicides present within water bodies. These studies provide valuable insights into the environmental impact of herbicides. For example, the mean S-metolachlor concentration in the sediments from the cultivated areas of the Ebro River in Spain was 0.33 ng × g^−1^ [9], which is similar to the value documented in the study by Yi-hao Qin et al.—0.49 ng × g^−1^ [10]. In a study conducted by Vera-Candioti et al., metolachlor was identified as the second most prevalent detected pesticide in surface and groundwater samples collected from agricultural areas in the Pampas region of Argentina. This analysis was carried out between November 2016 and May 2018 [11]. In a different study, several herbicides were examined. The analysis of river water samples from European countries revealed the presence of 2,4-dichlorophenoxyacetic acid (2,4-D) in 52% of the samples, while atrazine was detected in 68% of the samples and isoproturon in 70%. Notably, the research was conducted in autumn, which is an uncommon application period for herbicides. Consequently, these compounds were detected in relatively low concentrations [12]. Nevertheless, a separate study revealed that the concentration of metolachlor in winter was comparable to that measured during rain events, which can be attributed to the prolonged degradation time of this herbicide [13].

The extent of herbicide transport within a given environment is contingent upon a number of factors, including the degree of herbicide persistence and mobility, the application rate, meteorological conditions, precipitation, and topographical features [14]. The movement of pesticides to diverse environmental areas may occur via runoff erosion, volatilisation, and leaching. It is important to note that transport by runoff and leaching has the potential to result in the contamination of surface water and groundwater [15]. The contamination of surface and ground waters is a significant issue, given their utilization as drinking water supplies. Pesticide contamination of irrigation water has the potential to pollute agricultural farms in areas where they have not been applied. The quality of the ground or the drinking water beneath agricultural areas may be subject to degradation due to the irrigation of water contaminated with pesticides [16].

In addition to pesticide contamination, other compounds such as pharmaceuticals, surfactants, hormones, industrial chemicals, and heavy metals have also been detected in water [17]. The presence of a wide variety of potentially harmful substances in water resources has led to the formation of complex chemical mixtures, so-called chemical cocktails, which have been identified as a significant contributing factor to the degradation of water quality. Furthermore, the similarity of these compounds in terms of molecular weights often renders their separation and identification a very challenging task [18]. Chromatographic techniques, including liquid and gas chromatography, in combination with a variety of detection methods, like GC-MS, LC-MS/MS, UPLC-QTOFMS, and GC-ECD, are utilized for the analysis of trace quantities of pesticides in water. These methods are time-consuming [19], costly, and their implementation necessitates the expertise of skilled personnel [20].

Molecularly imprinted polymers are substances that exhibit a specific recognition ability for target pollutants, thereby improving the limit of detection and removal efficiency [21]. The process of molecular imprinting involves the polymerization of functional monomers in the presence of a template molecule, resulting in the formation of MIPs. Following the process of polymerization, the template is removed. As a result, the cavities that are complementary in terms of functional groups, shape, and size to the target molecule are formed on the polymer matrix [22]. MIPs exhibit both chemical and mechanical stability in the presence of extreme chemical and physical conditions. MIPs were selected for application in analytical separation and purification processes, utilizing techniques such as solid-phase extraction and chromatography due to their notable advantages [23].

In this paper, pH-sensitive *core–shell* polymers selective to S-metolachlor have been applied in the detection and separation of S-metolachlor herbicide in water samples from the river Stobrawa and a pond that are near cornfields in Poland. This work fully characterizes the sorbent described in our previous study [24]. The sorbent is then employed as a deposit within the SPE column for real sample applications.

## 2. Results and Discussion

### 2.1. Kinetics of Adsorption

The kinetics curves of S-metolachlor adsorption onto CS-MIP and CS-NIP particles were investigated using several kinetic models. The obtained data are summarized in Table 1 and Table 2.

It was concluded from the kinetic studies that sorption of S-metolachlor occurs in accordance with the *pseudo-first-order model* for both CS-MIP and CS-NIP. Nevertheless, the maximum sorption capacity of the sorbents is considerably lower than the experimental one. Furthermore, the sorption value for CS-NIP is higher than that for CS-MIP. In our previous work [25], we described a block molecularly imprinted polymer with a composition very similar to that of the CS-MIP under consideration. The kinetics of adsorption for the block MIP and NIP occurred according to the *pseudo-second order model*. However, in order to ascertain which type of kinetics of adsorption is decisive for CS-MIP and CS-NIP, Fick’s law was employed.

The analysis of the coefficients of determination enables the determination of which diffusion mechanisms are the most suited to the sorbent obtained. It is observed that the particle diffusion model is the optimal mechanism for controlling the sorption process for CS-MIP (R^2^ = 0.988), while the film diffusion model is more suitable for CS-NIP (R^2^ = 0.925), although the distribution coefficient is not close to one. The parameters $k_{a}$ and $k_{b}$ are used to ascertain which process occurs faster. It has been demonstrated that, in both cases of CS-MIP and CS-NIP, film diffusion is the faster of the two diffusion processes, which is a result of the presence of a thin polymer layer applied to the core particle.

### 2.2. Sorption Isotherms

In order to characterize the sorption process in complete detail, the sorption isotherms of S-metolachlor were investigated using four isotherm models. The following theories were considered: Langmuir, Freundlich, Dubinin–Radushkevich, and Scatchard isotherm models. The data obtained from this study are presented in Table 3.

Initially, the data were matched to the Langmuir isotherm model. The data obtained indicates an R^2^ coefficient for CS-NIP of 0.996. This could initially be interpreted as evidence that sorption occurs in accordance with this model. However, it was observed that the maximum sorption values for both sorbents (CS-MIP and CS-NIP) were found to be 50–140 times higher than the experimental values. Consequently, it was determined that this model was not applicable to either sorbent. In the Freundlich model, the parameter 1/*n*, which is indicative of surface heterogeneity, is higher for CS-NIP, indicating a more homogeneous surface compared to the surface of CS-MIP. Moreover, it is observed that the determination coefficient for both sorbents is equal to 0.999. Nevertheless, the value of $K_{F}$ (0.611 mg × g^−1^) related to adsorption capacity is much higher for CS-NIP than the experimental one (0.265 mg × g^−1^). For CS-MIP, the R^2^ (0.999) and $K_{F}$ (0.698 mg × g^−1^) results are very promising. However, it should be noted that CS-MIP has a heterogeneous surface; therefore, the value of 1/*n* should be higher than 1. The Dubinin–Radushkevich model was further considered. The value of $K_{DR}$ , which is constantly related to the free energy, is found to range from 1 to 8 kJ × mol^−1^ for both CS-MIP and CS-NIP. This indicates the physical mode of sorption. Furthermore, the maximum sorption calculated from this model is comparable to the experimental one. In addition, the determination coefficient is found to be 0.962. This indicates that the Dubinin–Radushkevich model can be utilized to characterize the sorption process of CS-NIP. In the case of CS-MIP, the value of R^2^ is equal to 0.907, and the maximum sorption capacity is slightly lower than the experimental one (0.425 mg × g^−1^). Therefore, this model was not selected. Finally, the Scatchard isotherm model was investigated. CS-MIP revealed two types of curves that were obtained, indicating the presence of two types of binding sites. For both curves, the determination coefficient is high, as well as the number of high-affinity binding sites, which is significantly higher than the number of low-affinity binding sites. Moreover, in the case of CS-NIP, low-affinity binding sites are not present. Therefore, it was determined that the Scatchard isotherm model would be the most suitable for CS-MIP.

### 2.3. Desorption Study

**Figure 1:**
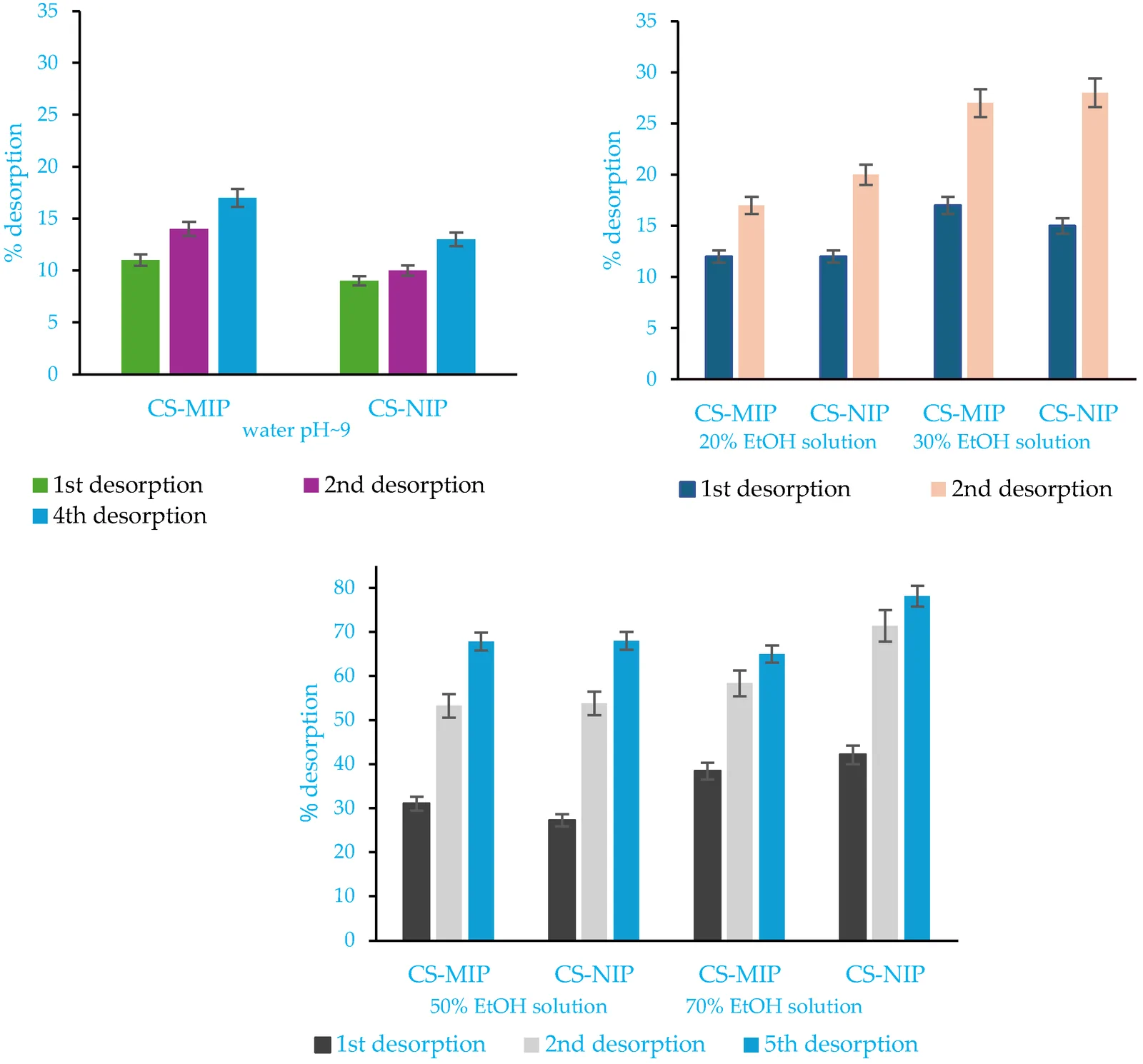
The desorption of S-metolachlor from a single-component solution.

In our previous work [24], we optimized the parameters of dynamic sorption on SPE columns and determined that the same material may be utilized up to three times, yielding an average regeneration factor of 96%. This study will focus on developing desorption procedures in detail. Initially, the conditions for the desorption of S-metolachlor from a single-component solution were selected. The desorption process was examined using pure water with a pH~9 and aqueous ethanol solutions with concentrations of 20, 30, 50, and 70% (pH~9). The data obtained are presented in Figure 1.

**Figure 2:**
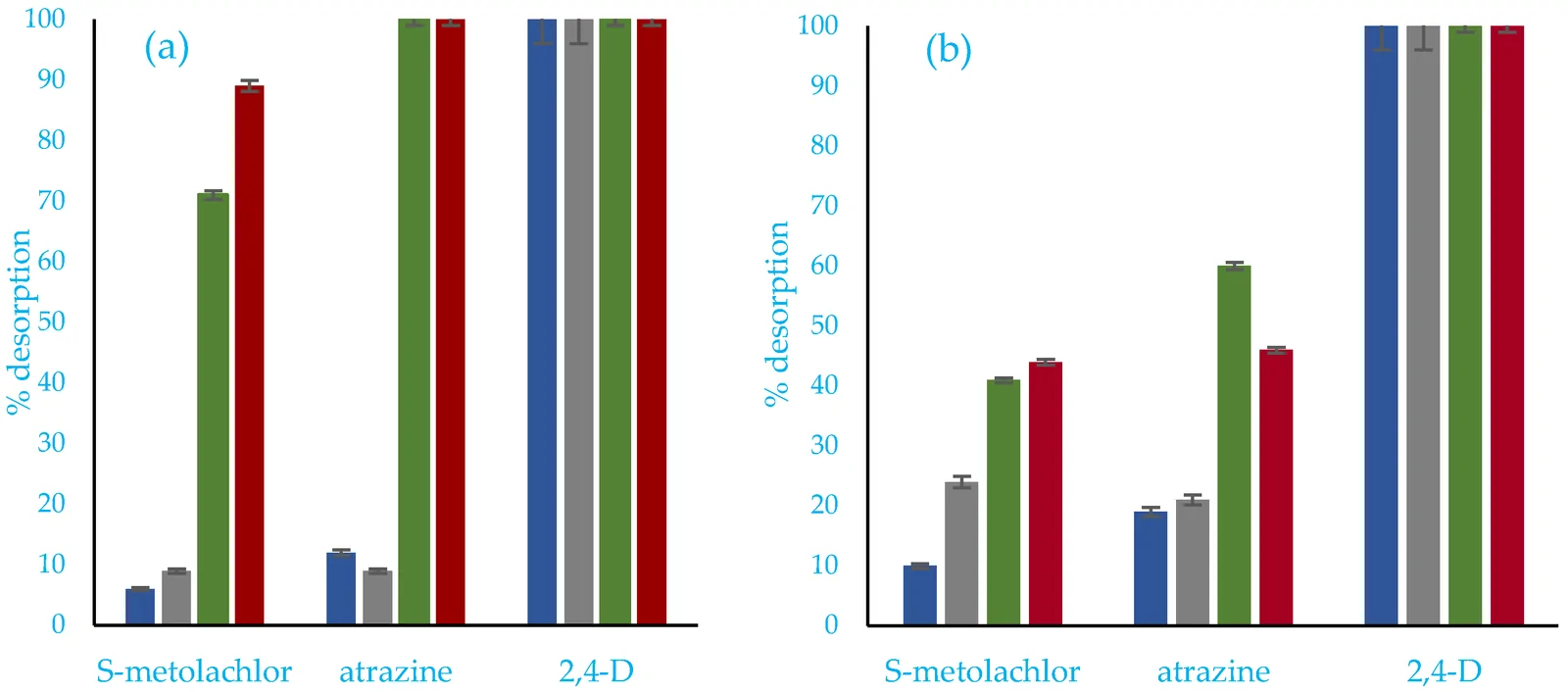
The desorption study: (**a**) First desorption using water with pH~9, and second and third desorption with 100% ethanol. (**b**) Desorption using 10% ethanol. 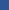 CS-MIP first desorption; 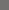 CS-NIP first desorption; 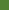 CS-MIP sum of first, second, and third desorption; and 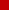 CS-NIP sum of first, second, and third desorption.

**Figure 3:**
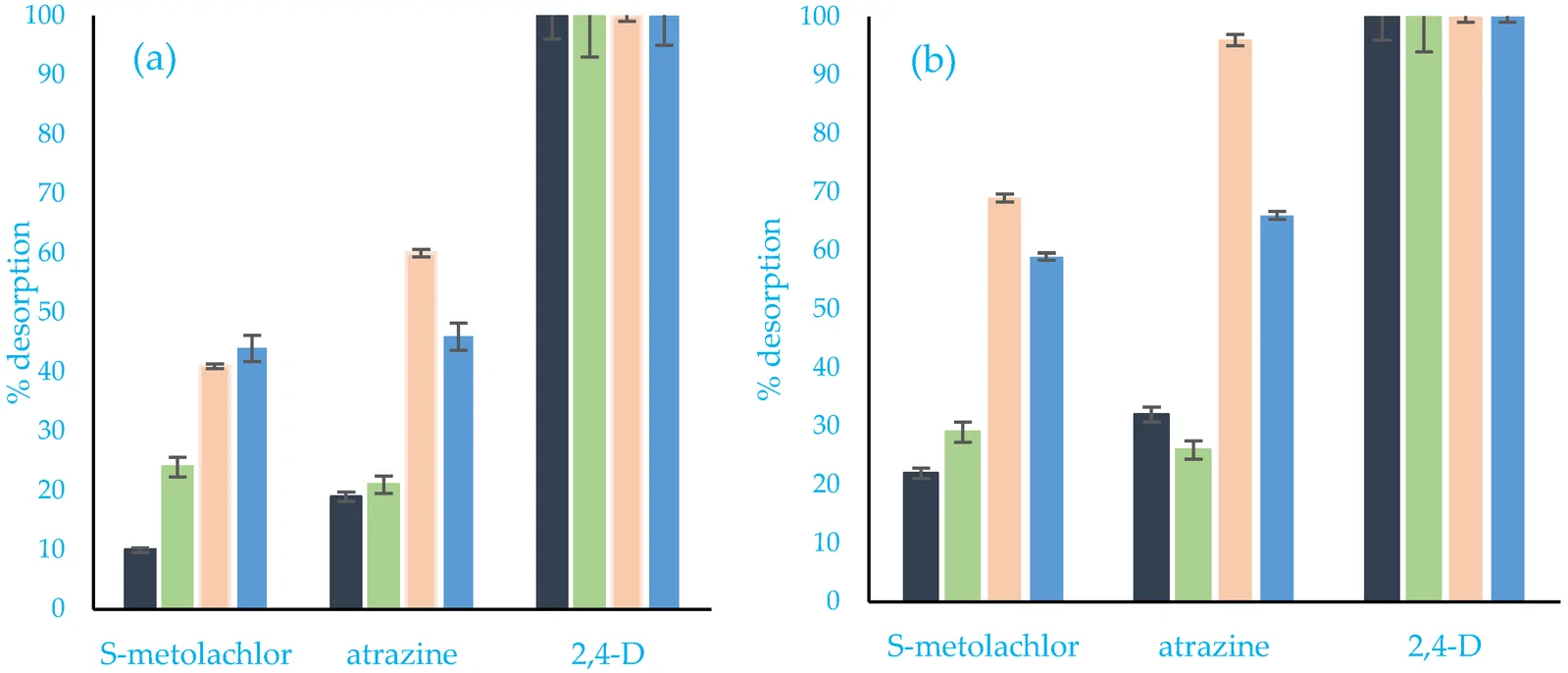
The desorption data: (**a**) Desorption using 20% ethanol; (**b**) Desorption using 30% ethanol. 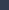 CS-MIP first desorption; 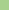 CS-NIP first desorption; 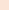 CS-MIP sum of first, second, and third desorption; and 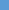 CS-NIP sum of first, second, and third desorption.

**Figure 4:**
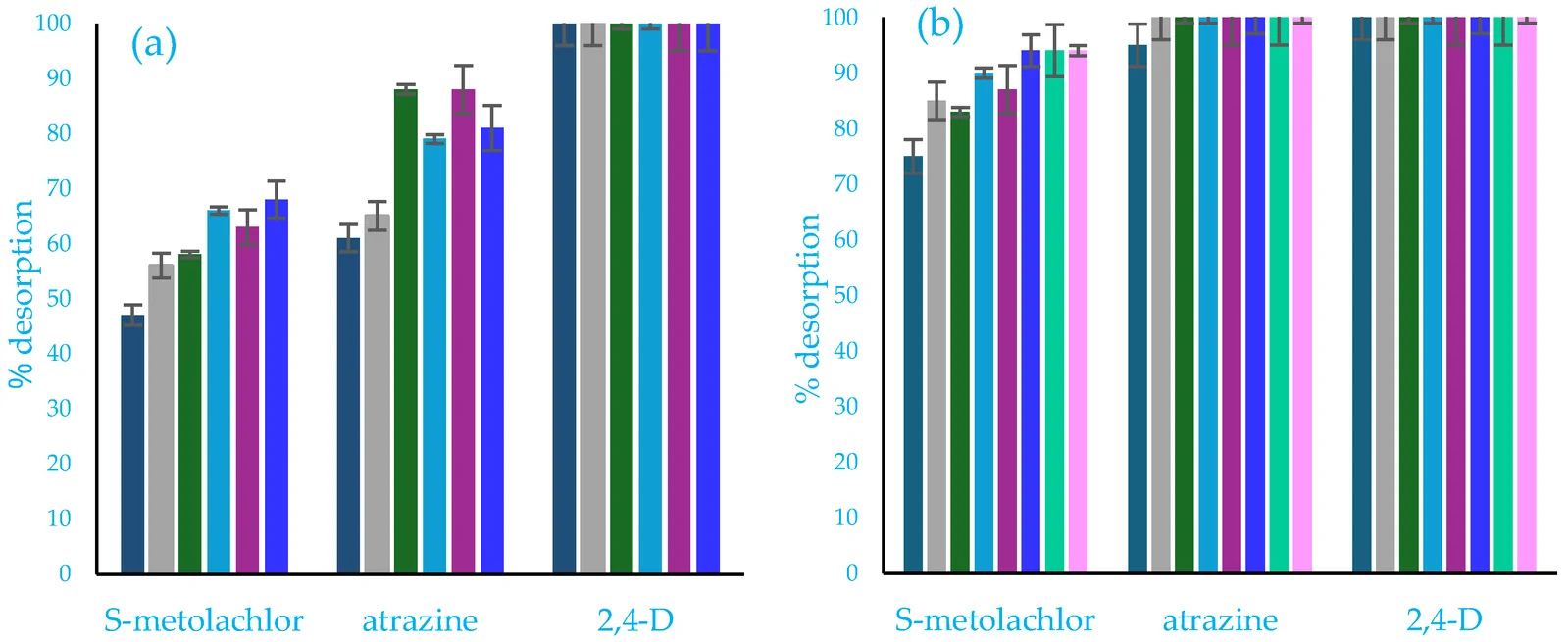
The desorption data: (**a**) first desorption using 40% ethanol, second desorption with 20% ethanol, and third desorption using water with pH~9; (**b**) Desorption using 100% ethanol. 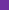 CS-MIP first desorption; 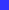 CS-NIP first desorption; 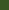 CS-MIP second desorption; 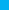 CS-NIP second desorption; 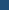 CS-MIP third desorption; 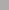 CS-NIP third desorption; 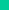 CS-MIP fourth desorption; and 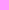 CS-NIP fourth desorption.

Acrylic acid is a pH-sensitive polymer. According to the results of our previous research and experience [25], water with a pH~9 was utilized for desorption. However, this eluent did not yield the expected results. The four-step process resulted in only 17% of S-metolachlor being desorbed. Consequently, the addition of ethanol was incorporated into further tests. The most suitable eluent was found to be a 50% aqueous ethanol solution with a pH~9. Two desorption cycles using this eluent resulted in the desorption of approximately 50% of the adsorbed herbicide molecules. However, further studies on the desorption of S-metolachlor from a multicomponent matrix were conducted in order to enable the utilization of the sorbent in real samples. The most challenging aspect of the study was the selection of an eluent capable of removing the residual herbicides while maintaining the S-metolachlor within the cavities. The various approaches were tested: 10%, 20%, 30%, 40%, and 100% aqueous solution of ethanol (pH~9) and pure water at pH~9. The results are presented in Figure 2, Figure 3 and Figure 4.

The tests were repeated on several occasions. The analysis of the data obtained indicates that the most effective eluent, which resulted in the almost complete removal of other herbicides present in the sample, while S-metolachlor remained bound in the pores, was 30% aqueous ethanol solution (pH~9). Following three desorption cycles, atrazine and 2,4-D desorbed 96% and 100%, respectively, while S-metolachlor desorbed 69%. However, in order to desorb almost all of the adsorbed S-metolachlor and clean the bed, four desorption cycles with pure 100% alcohol are required.

### 2.4. River and Pond Water Characteristics

The study examined water samples from two distinct locations: the Stobrawa River and a pond situated in proximity to cornfields in the Opolskie Province of Poland. An analysis was conducted on the water samples obtained from both the river and the pond with the objective of evaluating their ionic and physical properties. The tests were performed in its natural state, as well as undergoing treatment with microfiltration and ultrafiltration. The examination of the content of fluorides, chlorides, nitrates (V), and sulfur (VI) was conducted through the process of ionic analysis. The values of these compounds (see Table 4) decreased after each purification stage, but were not significantly lower.

**Figure 5:**
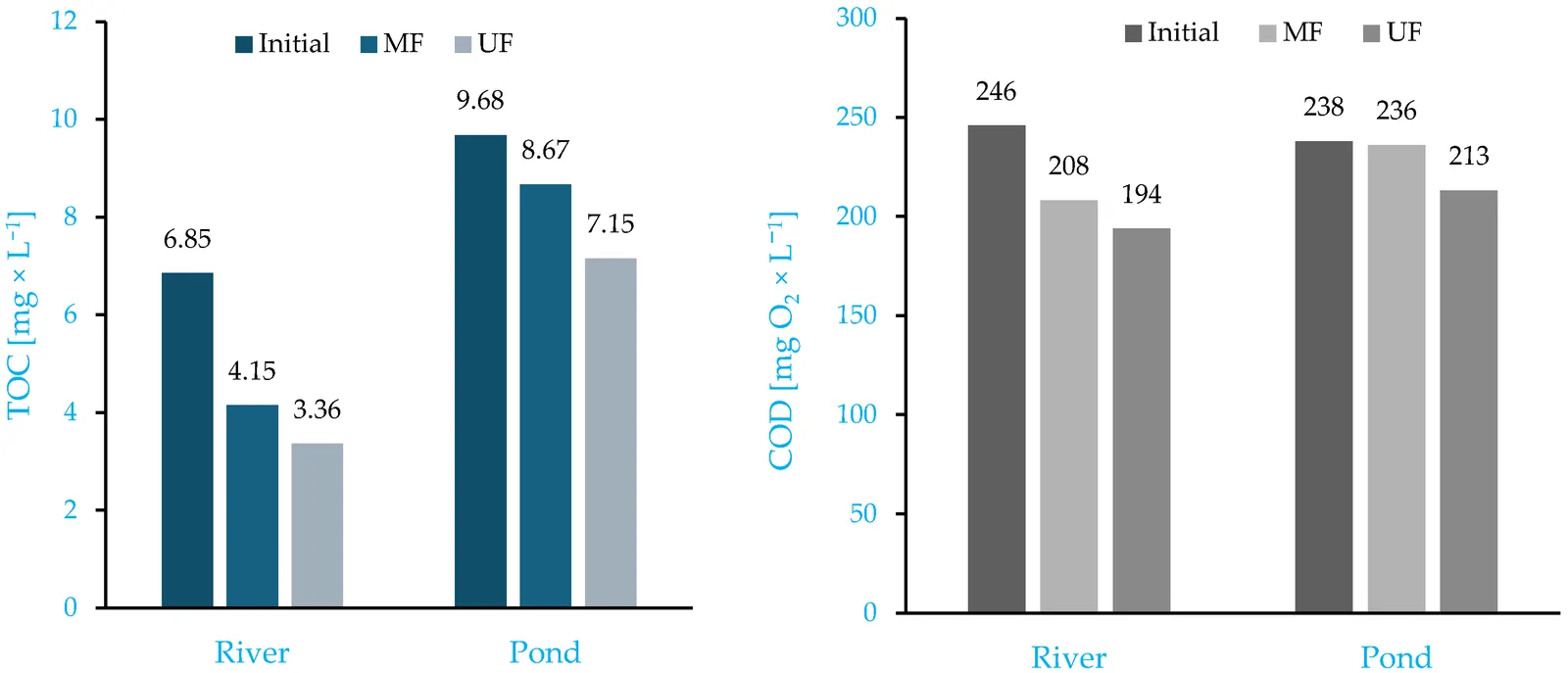
Analysis of Total Organic Carbon (**left**) and Chemical Oxygen Demand (**right**).

A further analysis was conducted on the Total Organic Carbon and Chemical Oxygen Demand, the values of which are presented in Figure 5. In accordance with the stipulations outlined in the Regulation of the Minister of Infrastructure of Poland [26], the maximum permissible concentration of Total Organic Carbon in a lowland river, such as the Stobrawa, is set at a value below 8.2 mg × L^−1^ for I class surface water quality. The result of the conducted tests indicates a concentration of 6.85 mg × L^−1^, falling within the prescribed parameters. However, the determined COD value significantly exceeds the parameters even for Class II surface water quality. The aforementioned regulation does not include water from ponds.

Subsequently, a series of physical properties was examined, including conductivity, turbidity, absorbance, pH, and color (see Table 5). The measurement of the absorbances was conducted at a wavelength of 254 nm, facilitating the assessment of water contamination in relation to the presence of organic compounds. As detailed in regulation [26], the specific conductivity value for water quality class I is less than 420 μS × cm^−1^. However, the value obtained through the test conducted is 421.1 μS × cm^−1^, which is in proximity to this threshold. It is important to note that the remaining parameters specified are not incorporated within the regulation. The turbidity and color values decrease with each subsequent stage of purification. The river water exhibited increased transparency at the initial stage, while the pond water demonstrated significantly elevated levels of turbidity. Therefore, for the river water sample, a slight decrease in color value was observed; however, a particularly significant change in color and turbidity was observed for the water sample from the pond after the ultrafiltration process. In consideration of the data presented, it can be deduced that the water of the Stobrawa River exhibits characteristics of Class I water quality for specific parameters. For other parameters, it demonstrates characteristics of lower classes, thus indicating that the water can be characterized as medium quality.

### 2.5. Real Sample Detection

In our previous study [24], we examined the simulated real-one conditions by testing a tap water solution of S-metolachlor. However, the composition of tap water is less complex than that of samples taken from rivers or ponds. The standard SPE procedure was utilized for the detection of S-metolachlor in both types of samples. However, this procedure did not reveal the presence of the herbicide in the water. Consequently, a methodology was designed to concentrate the S-metolachlor in the water. This was achieved by passing 50 mL of water from the river and the pond through a column. However, the test also did not detect the presence of S-metolachlor in the water samples. S-metolachlor was added to the collected water samples that were used in this study, thus creating a simulated herbicide system in real samples, which was then subjected to further testing.

#### 2.5.1. Analysis of Water from the River and Pond

S-metolachlor (concentration ~35 mg × L^−1^) was added to the untreated water sample in order to determine whether the sorbent would be capable of catching herbicide molecules in the presence of other interference compounds. The water was also purified by membrane filtration through a 200 and 20 nm PP membrane. S-metolachlor (concentration ~35 mg × L^−1^) was then added to the purified water, and the sorption process was carried out. The results are presented in Table 6.

**Figure 6:**
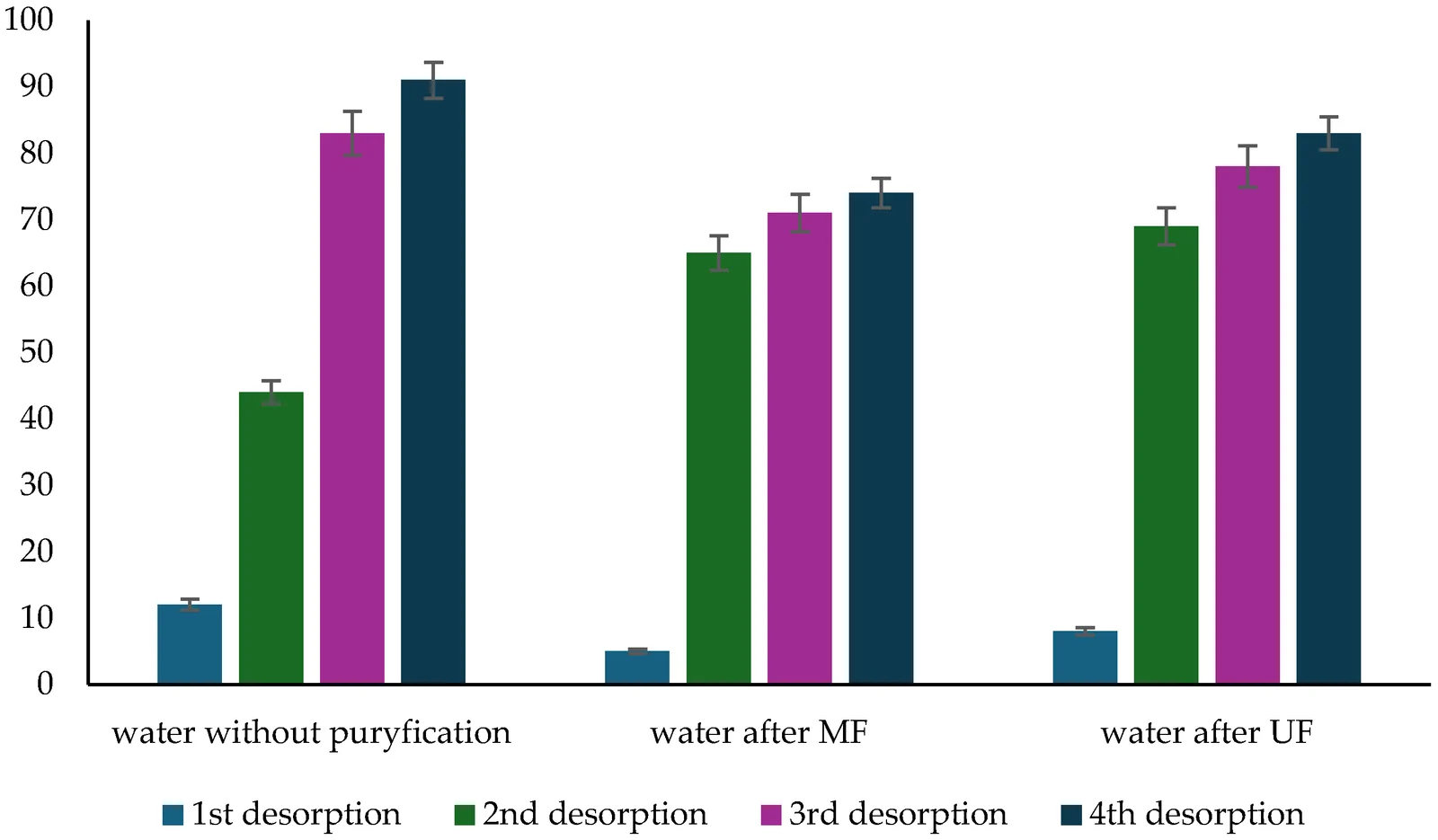
Desorption of S-metolachlor from river samples.

**Figure 7:**
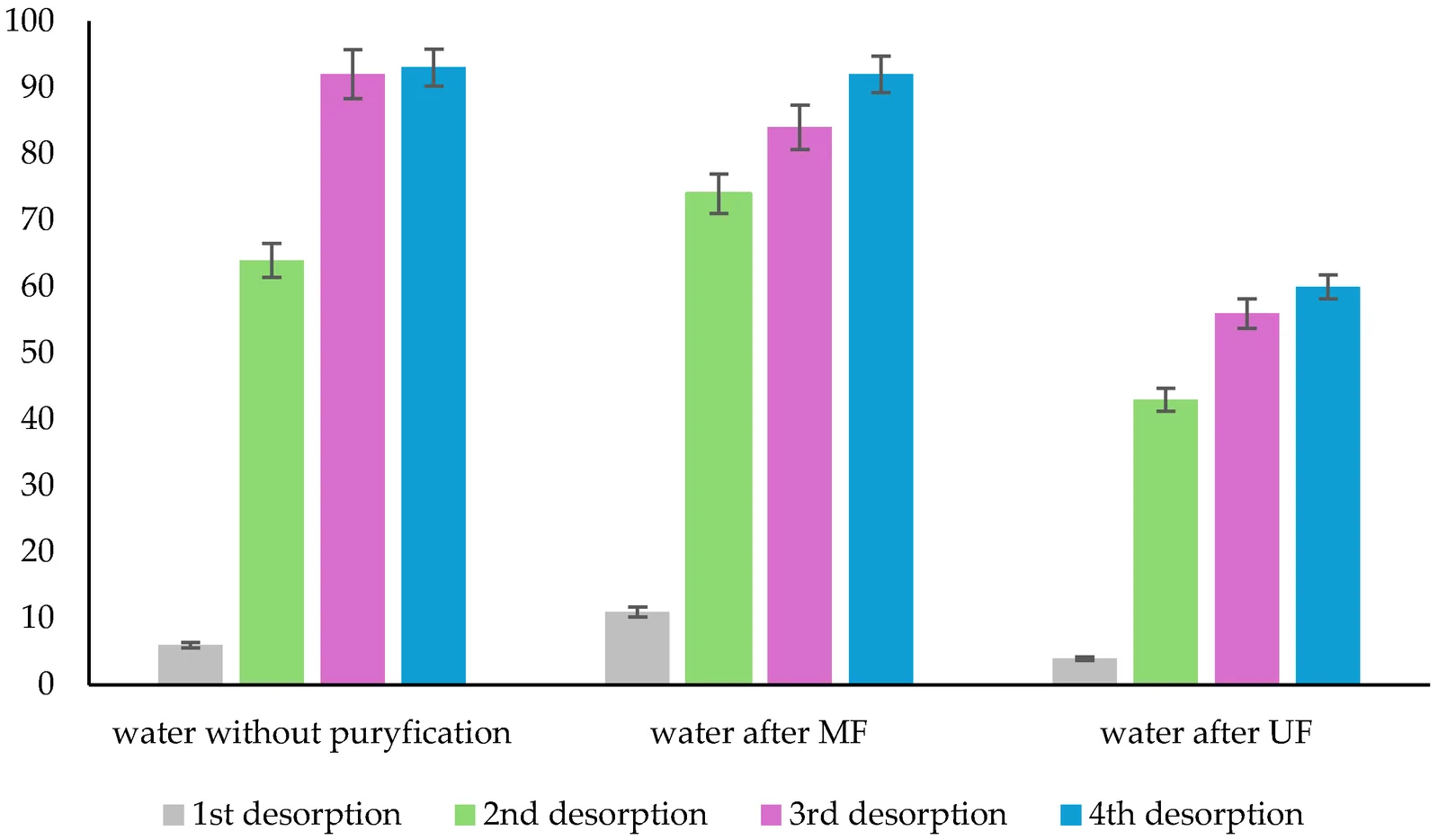
Desorption of S-metolachlor from pond samples.

The above data show that, for river samples, the membrane purification process does not influence the sorption capacity of S-metolachlor by molecularly imprinted polymers. The average sorption value was the same in all three samples, equating to 0.530 mg × g^−1^. This proves that the selective sorption of S-metolachlor does not require membrane filtration. The values obtained from the river samples are higher than those obtained from model solutions, which may be due to the higher ionic strength. In the case of water samples from a pond, the sorption values are also higher than those obtained from model solutions. With subsequent water purification, the sorption value for both sorbents (CS-MIP and CS-NIP) increases, and the difference between the values obtained for CS-MIP and CS-NIP decreases. This phenomenon may be attributed to the initial high turbidity and color values, indicating high pollution, which reduced marginally following microfiltration but increased considerably after the ultrafiltration process. Subsequent to each treatment stage, a reduced number of interfering compounds was observed in the water, thereby yielding increased sorption values. However, in the case of pond water, the sorption process of S-metolachlor can also be successfully carried out without purifying the sample because the sorption value is high enough to detect potential contamination with S-metolachlor. In the following stage of the experiment, the adsorbed S-metolachlor molecules undergo desorption, with the results illustrated in Figure 6 and Figure 7.

The desorption process was carried out in accordance with the developed procedure. Desorption was performed on all types of samples, including river water and pond water, both before and after treatment. The initial desorption was conducted with distilled water, having a pH of approximately 9. The subsequent three desorptions were carried out using 100% ethanol, with the objective of completely removing all adsorbed molecules. The initial desorption process was designed to eliminate contaminants, achieving an average desorption rate of 8% for S-metolachlor in river water samples and 7% for the herbicide in pond samples. With regard to river water samples, the second desorption process proved to be the least effective for untreated water (only 44%), and for samples after membrane treatment, an average of 67% was achieved. However, the desorption process for untreated samples was the most successful, with over 90% of the herbicide molecules desorbed. In contrast, for samples treated with 200 and 20 nm membranes, 74 and 83% were desorbed, respectively. This outcome confirms the previously proposed thesis that the sorption and desorption of S-metolachlor from complex water matrices can be achieved without the necessity of prior sample preparation. In the case of water samples from the pond, S-metolachlor desorbed the least favorably from samples after filtration through a 20 nm membrane, purifying the deposit by only 60%. As in the case of river water samples, the desorption process was most favorable in samples without prior purification, regenerating the deposit by 93%. The data presented indicate the efficacy of the sorbent in analyzing environmental samples without the necessity for prior purification.

#### 2.5.2. The Permeate Flux

**Figure 8:**
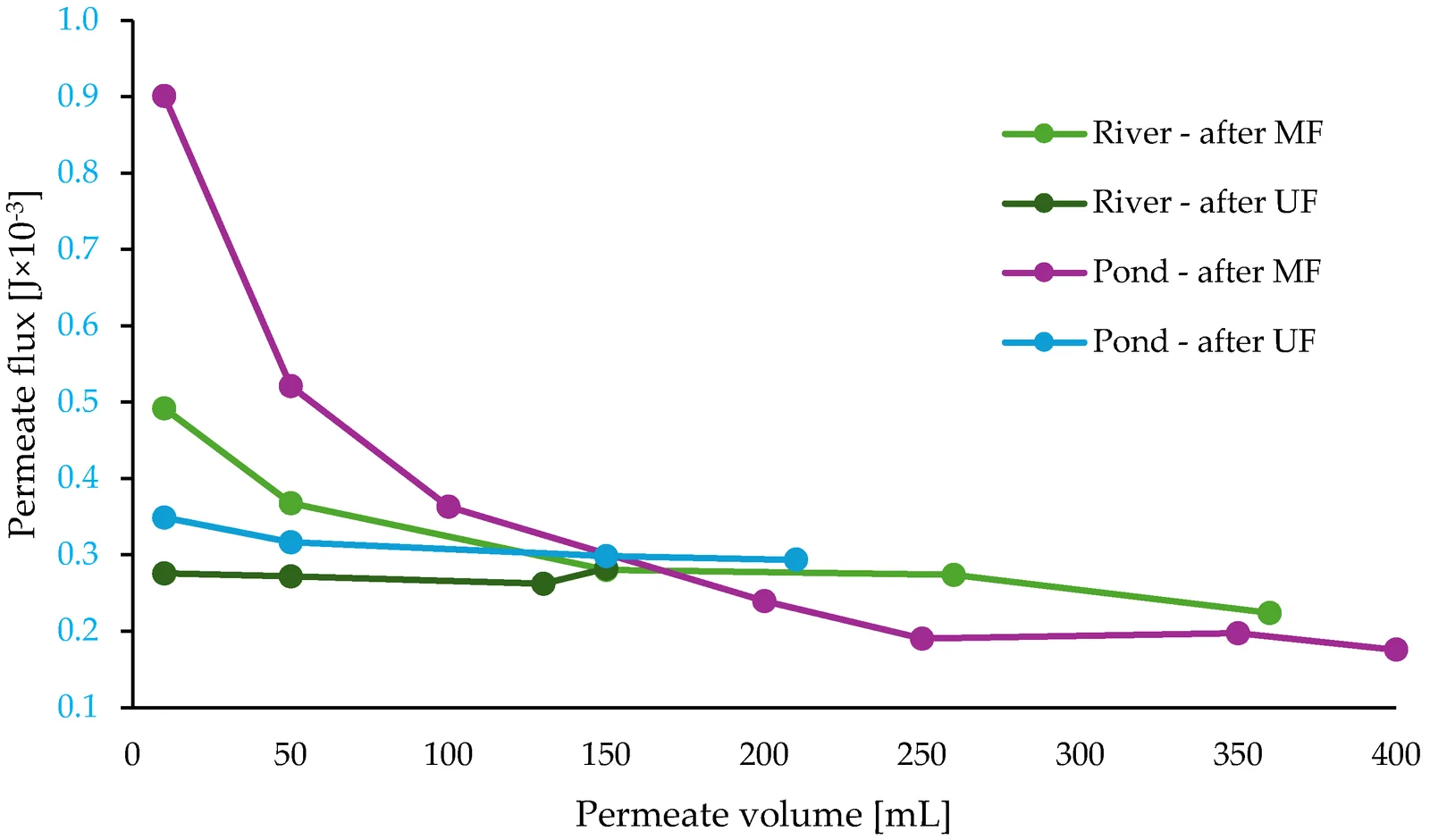
The permeate flux for water from the river and the pond.

The flux was measured in relation to the increasing filtered volume of permeate through the membrane. The results obtained are presented in Figure 8. The data collected demonstrates a direct correlation between the increase in permeate volume and the resulting decrease in permeate flux.

## 3. Materials and Methods

### 3.1. Reagents and Chemicals

The following agents were used without any further treatment. Acrylamide (AA) was purchased from Thermo Scientific Chemicals (Warsaw, Poland), and Acrylic acid (AAc) was obtained from Acros Organics (Warsaw, Poland). Ammonium persulfate (APS), N, N′-methylenebis (acrylamide) (BIS), and N, N, N′, N′-tetramethylethylenediamine (TEMED) were received from Sigma-Aldrich (Warsaw, Poland). S-metolachlor (SMCh) was sourced from NOVAGRA (Błonie, Poland). Atrazine was acquired from ABCR GmbH and Co. (Karlsruhe, Germany), and 2,4-dichlorophenoxyacetic acid (2,4-D) was supplied from Corteva (Indianapolis, IN, USA). Sodium hydroxide (NaOH) was purchased from POCH S.A. (Gliwice, Poland). Poly(vinyl chloride) (Ongrovil^®^ S-5167) was procured from BorsodChem (Kazincbarcika, Hungary), with medium molecular weight, and with a K value (an indicator of molecular weight) of 66–68. High-performance liquid chromatography (HPLC) grade acetonitrile (ACN) and ethyl alcohol (EtOH) were purchased by POCH S.A. (Gliwice, Poland). Ultrapure water was obtained with a Milli-Q System (Merck Millipore, Warsaw, Poland).

### 3.2. Preparation of Core–Shell Molecularly Imprinted Polymers

The synthesis of the *core–shell* molecularly imprinted polymer was conducted in accordance with the procedure delineated in our previous work [24]. The experimental process involved the contact of approximately 5 g of poly(vinyl chloride) grains, which had undergone the amination process, with 14 mL of an aqueous solution of the prepolymerization mixture. The mixture consists of the two functional monomers: acrylic acid (5.75 mmol) and acrylamide (6.96 mmol), as well as the cross-linking agent N, N′- methylenebis(acrylamide) (4.93 mmol) and S-metolachlor (0.40 mmol). The prepolymerization mixture was then subjected to sonication for a period of 10–15 min, with the objective of achieving the dissolution of BIS. Subsequently, 15.3 mL of water was added to the reactor, after which the mixture was left to incubate for an additional 10–15 min. In the following stage of the process, the prepolymerization mixture was purged with nitrogen for a period of 5–10 min. Subsequently, ammonium persulfate (0.30 mmol) and tetramethylethylenediamine (0.23 mmol) were introduced as an initiator. The reaction was carried out for 24 h at ambient temperature. The mixture was then transferred to a Buchner funnel and extracted on a Soxhlet apparatus for 24 h. Subsequently, the polymers were dried at room temperature. The non-imprinted core–shell polymer (CS-NIP) was synthesized in an identical method to CS-MIP, with the exception that S-metolachlor molecules were not incorporated during the process.

### 3.3. Adsorption Experiments

#### 3.3.1. Dynamic Mode Sorption on the SPE Columns

In the case of all dynamic mode sorption tests carried out on the SPE columns, approximately 0.05 g of polymers (CS-MIP and CS-NIP) was inserted into the 3 mL SPE columns. Thereafter, samples were subjected to a water wash in order to activate the surface. Subsequently, 2 mL of S-metolachlor solution was added, and the sorbents were incubated for varying periods of time. Subsequently, the concentration of the solution following sorption was ascertained by high-performance liquid chromatography. The calculation of sorption was then performed in accordance with the following equation:

(1) $$q=\;\frac{\left(C_{0}-C_{eq}\right)\times V\;}{m}$$

where $C_{0}$ is the initial concentration of S-metolachlor (mg × L^−1^), $C_{eq}$ is the equilibrium concentration of S-metolachlor (mg × L^−1^), V is the volume of the solution (L), and m is the mass of the polymer (g).

#### 3.3.2. Kinetics of Adsorption

Investigations into the kinetics of adsorption were conducted using a 250 mL S-metolachlor solution (concentration 30 mg × L^−1^) and a mass of 2.5 g of the sorbent (CS-MIP and CS-NIP). The experiment was carried out at room temperature, and the concentration of the samples was measured at different time intervals (from 5 to 180 min) using HPLC methods. The *pseudo-first-order* and *pseudo-second-order* models, as well as Fick’s law, were employed to ascertain the key process parameters.

##### Pseudo-First-Order Model

In the *pseudo-first-order model*, the difference between the amount of adsorbed adsorbate molecules on the adsorbents at equilibrium adsorption time and a defined time is determined by the adsorption process rate. In this model, the process of adsorption is independent of the nature of the adsorbates. The linear form of the pseudo-first-order model is presented below in the form of Equation (2). The value of the rate constant $k_{1}$ was determined through calculation based on the plot $log\left(q_{eq}-q_{t}\right)$ versus time.

(2) $$\log\left(q_{eq}-q_{t}\right)=\log\left(q_{eq}\right)-\frac{k_{1}t}{2.303}$$

where $k_{1}$ is the sorption rate constant (1 × min^−1^) and $q_{eq}$ and $q_{t}$ are the mass of S-metolachlor adsorbed at the equilibrium time and at time t , respectively (mg × g^−1^) [27,28].

##### Pseudo-Second-Order Model

In the *pseudo-second-order model*, it is assumed that the sharing or exchanging of electrons between the adsorbent and the adsorbate is a rate-limiting step. This kinetics model postulates that the adsorption process is controlled by chemisorption. The following Equation (3) is used to describe the linear form of the pseudo-second-order model:

(3) $$\frac{t}{q_{t}}=\frac{1}{k_{2}q_{eq}^{2}}+\frac{1}{q_{eq}}t$$

where $k_{2}$ is the sorption rate constant (g × (mg × min)^−1^) and $q_{eq}$ and $q_{t}$ are the mass of S-metolachlor adsorbed at equilibrium time and at time t , respectively (mg × g^−1^) [28,29].

##### Fick’s Second Law

To ascertain whether particle or film diffusion is the rate-limiting process, Fick’s second law was employed to determine the diffusion mechanisms. The kinetics of the sorption process controlled by diffusion through the film are expressed as follows (4)

(4) $$k_{a}t=-ln(1-\frac{q_{t}}{q_{eq}})$$

where $k_{a}$ is the sorption rate constant (1 × min^−1^) and $q_{t}$ and $q_{eq}$ are the amount of S-metolachlor adsorbed at time t and at the equilibrium time, respectively (mg × g^−1^).

The particle diffusion-controlled adsorption process is estimated by Equation (5). The sorption rate constant $k_{b}$ was calculated from a plot of $-ln(1-{\left(\frac{q_{t}}{q_{eq}}\right)}^{2})$ versus time [30].

(5) $$k_{b}t=-ln(1-{\left(\frac{q_{t}}{q_{eq}}\right)}^{2})$$

where $k_{b}$ is the sorption rate constant (1 × min^−1^) and $q_{t}$ and $q_{eq}$ are the amount of S-metolachlor adsorbed at time t and at the equilibrium time, respectively (mg × g^−1^) [30].

#### 3.3.3. Sorption Isotherms

The interaction between adsorbent and adsorbate in the aqueous solution is explained by uptake isotherms. The data obtained from the sorption study were fitted to four linear adsorption isotherm models: Langmuir, Freundlich, Dubinin–Radushkevich, and Scatchard. These models are the most well-known and frequently employed, so we ascertained whether any of them would also align with the experimental data presented.

##### Langmuir Model

The Langmuir isotherm is based on the assumption that all binding sites on the surface are equivalent, thus demonstrating the relationship between the amount of bound analyte and the amount of free analyte in the system at the equilibrium. The mathematical linear form of the Langmuir model can be expressed as follows (6):

(6) $$\frac{1}{q_{e}}=\;\frac{1}{q_{m}K_{L}C_{e}}+\;\frac{1}{q_{m}}$$

where $q_{e}$ is the absorption capacity at equilibrium (mg × g^−1^), $q_{m}$ is the maximum adsorption capacity (mg × g^−1^), $K_{L}$ is the Langmuir parameter—the affinity of the adsorption sites (L × mg^−1^)—and $C_{e}$ is the equilibrium concentration of S-metolachlor (mg × L^−1^) [31].

##### Freundlich Equation

The Freundlich isotherm model is based on the sorption on a heterogeneous surface and the interaction between adsorbed molecules. In the case of a slope value between 0 and 1, it measures the surface heterogeneity or the intensity of adsorption. The slope value is indicative of the type of material: if it is close to 1, the material is homogeneous, and if the calculated value is higher than 1, the material is heterogeneous. The mathematical linear form of the Freundlich model is given by the following Equation (7):

(7) $$logq_{e}=\frac{1}{n}\;logC_{e}+logK_{F}\;$$

where $q_{e}$ is the absorption capacity at equilibrium (mg × g^−1^), $K_{F}$ is the Freundlich constant related to adsorption capacity (mg × g^−1^), n is a heterogeneity index, and $C_{e}$ is the equilibrium concentration of S-metolachlor (mg × L^−1^) [32].

##### Dubinin–Radushkevich Model

The Dubinin–Radushkevich model was investigated to estimate whether the adsorption process was chemisorption or physical adsorption. When the ε value is found to be in the range of 1–8 kJ × mol^−1^, this is indicative of physical adsorption. In the range of 8 to 16 kJ × mol^−1^, the sorption process is assumed to occur through ion exchange. However, for values exceeding 16 kJ × mol^−1^, the sorption process is deemed to be of a chemical character. The following Equation (8) represents the linear mathematical formulation of the Dubinin–Radushkevich model:

(8) $${lnq}_{e}={lnq}_{m}-\;{K_{DR}\varepsilon }^{2}$$

where $q_{e}$ is the absorption capacity at equilibrium (mg × g^−1^), $q_{m}$ is the maximum adsorption capacity (mg × g^−1^), $K_{DR}$ is the activity coefficient related to adsorption-free energy (mol^2^ × kJ^−2^), and ε is the Polanyi adsorption potential (kJ × mol^−1^) [32,33].

##### Scatchard Equation

The Scatchard isotherm model is a reliable method for determining the nature of active sites on the adsorbent. In the event of a linear fit being obtained from the Scatchard model, it can be deduced that the adsorbent contains one type of active site (homogenous surface). Whereas a non-linear fit will reveal that the adsorbent contains more than one type of binding site (heterogeneous surface). The mathematical linear form of the Scatchard model is given by the following Equation (9):

(9) $$\frac{q_{e}}{C_{e}}=PN-Pq$$

where $q_{e}$ is the absorption capacity at equilibrium (mg × g^−1^), $C_{e}$ is the equilibrium concentration of S-metolachlor (mg × L^−1^), *P* is the binding affinity (L × mmol^−1^), and N is the number of binding sites (mmol × g^−1^) [34].

#### 3.3.4. Bed Regeneration

The calculation of the regeneration factor ( $R_{f}$ ) of the deposit was undertaken using the following Equation (10):

(10) $$R_{f}=\;\frac{m_{e}}{m_{a}}\;\times 100\%$$

where $m_{e}$ is the mass of eluted S-metolachlor (mg), and $m_{a}$ is the mass of absorbed S-metolachlor (mg).

### 3.4. Membrane Process

The water samples from the river and pond were treated using a membrane filtration process. The microfiltration (MF) and ultrafiltration (UF) processes were conducted on Amicon Millipore cell model 8200, utilizing polypropylene (PP) membranes with an average pore size of 200 nm (MF) and 20 nm (UF), respectively, supplied by Celgard. The diameters of the membranes employed in the process were approximately 63 mm. The filtration process was carried out at an approximate pressure of 0.1 MPa.

#### Membrane Regeneration Process

Regeneration of the polypropylene membranes was conducted according to the procedure detailed in our previous work [35]. In summary, a 0.02 M NaOH solution was employed. Filtration of the alkaline solution through the membranes continued for a period of 10 min. Subsequently, the membranes were washed with distilled water.

### 3.5. Permeate Flux

The calculation of the permeate flux during filtration through the membrane was performed in accordance with the following Equation (11):

(11) $$J=\;\frac{v}{S\;\times \;t\;}$$

where v is the permeate volume (mL), *S* is the active surface area of the membrane (cm^2^), and t is the time of permeate collection (s).

### 3.6. Analytical and Characterization Methods

#### 3.6.1. High Performance Liquid Chromatography

A Shimadzu high performance liquid chromatograph model SCL-40 (Shim-Pol, Izabelin, Poland), equipped with a photodiode array (PDA) detector 40 (Shim-Pol, Izabelin, Poland), and a chromatographic column (3 μm ARION^®^ C18, 150 × 4.6 mm) (Shim-Pol, Izabelin, Poland), was used. For the analysis, 30 μL of sample solution was injected and eluted isocratically at a flow rate of 1 mL × min^−1^, using a mixture of water and acetonitrile (1:1, *v*/*v*) as a mobile phase. The temperature was kept at 30 °C, and the wavelength was set to 254 nm.

#### 3.6.2. Water Analysis

The concentration of bromides, fluorides, chlorides, nitrates (V), and sulphates (VI) was determined on a Thermo Scientific Dionex Aquion (Thermo Fisher Scientific, Warsaw, Poland) ion chromatograph with a conductometric detector. Determinations of sodium and potassium were conducted utilizing a Flame Photometer Model BWB-XP by MS Spectrum (BWB Technologies, Newbury, UK). UV absorption was measured using a Shimadzu UV-VIS 1800 spectrophotometer (Shim-Pol, Izabelin, Poland). pH and conductivity were determined utilizing a Digital Multimeter HQ40D (Hach Lange, Wrocław, Poland) with an IntelliCAL™ PHC 101 electrode (Hach Lange, Wrocław, Poland). The Chemical Oxygen Demand (COD) was estimated with the Bichromate method, and the Total Organic Carbon (TOC) was measured using the Hach IL550 carbon analyzer (Hach Lange, Wrocław, Poland).

## 4. Conclusions

The present study employs *core–shell* molecularly imprinted polymers for the detection of S-metolachlor in water samples collected from a river and a pond in proximity to cornfields in Poland. The purification of the water samples by membrane filtration, specifically microfiltration and ultrafiltration, did not enhance the sorption capacity of S-metolachlor by CS-MIP. A very important conclusion from this study is that the sorbent can be used for the detection and determination of S-metolachlor concentration from a complex water matrix without prior sample preparation, which significantly facilitates and shortens the analysis time. A method for the desorption of S-metolachlor from a multicomponent solution was also developed, thereby demonstrating that the optimum eluent is a 50% aqueous ethanol solution with a pH of approximately 9. The sorbents were also investigated to ascertain their sorption properties. The kinetics and isotherms of sorption were the focus of the study. The experimental results demonstrated that the kinetics for CS-MIP followed the particle diffusion model, while for CS-NIP, they exhibited a tendency to fit the film diffusion model. The Scatchard model was found to be the most appropriate isotherm model for CS-MIP, and the Dubinin–Radushkevich was found to be the most appropriate model for CS-NIP.

## Figures and Tables

**Table 1:** The result obtained from the calculation according to the pseudo-first and pseudo-second order models.

	Pseudo-First-Order Model			Pseudo-Second-Order Model		
	$k_{1}$ (1 × min^−1^)	R^2^	*q_max_*_1_ (mg × g^−1^)	$k_{2}$ (g × (mg × min)^−1^)	R^2^	*q_max_*_2_ (mg × g^−1^)
CS-MIP	0.005	0.984	0.096	0.129	0.949	0.517
CS-NIP	0.007	0.925	0.116	0.024	0.512	0.782

**Table 2:** The parameters obtained from the calculation according to Fick’s law.

	Film Diffusion		Particle Diffusion	
	$k_{a}$ (1 × min^−1^)	R^2^	$k_{b}$ (1 × min^−1^)	R^2^
CS-MIP	0.005	0.972	0.002	0.988
CS-NIP	0.007	0.925	0.003	0.854

**Table 3:** Isotherm models data for the S-metolachlor sorption process.

	Langmuir Model			Freundlich Model			Dubinin–Radushkevich Model			Scatchard Model					
	$K_{L}$	*q_m_*	$R^{2}$	$K_{F}$	1/n	$R^{2}$	$K_{DR}$	*q_m_*	$R^{2}$	$P_{1}$	$N_{1}$	$R_{1}^{2}$	$P_{2}$	$N_{2}$	$R_{2}^{2}$
CS-MIP	0.02	21.8	0.827	0.7	0.69	0.999	1.1	0.35	0.907	6.8	16.4	0.969	1.1	5.2	0.985
CS-NIP	0.32	36.2	0.996	0.6	0.71	0.999	1.0	0.36	0.962	-	-	-	0.6	3.4	0.943

**Table 4:** Ionic parameters of water from the river and the pond.

Type of Sample	Type of Purification	Flourides [mg × L^−1^]	Chlorides [mg × L^−1^]	Nitrates (V) [mg × L^−1^]	Sulfur (VI) [mg × L^−1^]	Na [mg × L^−1^]	K [mg × L^−1^]
River	Without	0.06	46.10	4.27	81.20	8.30	2.50
	MF	<0.02	45.50	4.20	80.10	6.10	2.00
	UF	<0.02	45.10	4.16	79.00	5.40	1.90
Pond	Without	0.30	52.00	19.01	103.40	1.00	6.80
	MF	0.14	50.90	6.18	100.90	0.50	6.70
	UF	0.13	50.30	5.90	98.10	0.03	5.90

**Table 5:** Analysis of physical properties of water.

Type of Sample	Type of Purification	Conductivity [μS × cm^−1^]	Turbidity [NTU]	Absorbance [cm^−1^]	pH	Color [mg Pt × L^−1^]
River	Without	421.1	9.87	0.031	8.28	17.83
	MF	432.9	6.60	0.021	8.41	17.52
	UF	346.6	2.17	0.015	8.33	15.07
Pond	Without	478.1	11.30	0.038	8.57	32.16
	MF	353.2	9.30	0.032	8.4	31.31
	UF	458.9	0.70	0.014	8.65	13.17

**Table 6:** The results of the real sample study.

	River Stobrawa		Pond	
	CS-MIP [mg × g^−1^]	CS-NIP [mg × g^−1^]	CS-MIP [mg × g^−1^]	CS-NIP [mg × g^−1^]
Water without purifying	0.530 ± 0.022	0.446 ± 0.058	0.448 ± 0.015	0.360 ± 0.038
Water after MF	0.530 ± 0.059	0.472 ± 0.088	0.494 ± 0.043	0.449 ± 0.027
Water after UF	0.530 ± 0.079	0.553 ± 0.064	0.657 ± 0.080	0.560 ± 0.077

## Data Availability

Data are contained within the article.
